# Perspectives of Hospitalists in an Academic Health System

**DOI:** 10.26502/aimr.0139

**Published:** 2022-11-14

**Authors:** TR Clarke, Josh Laban, Ahmed Luqman

**Affiliations:** Assistant Professor of Clinical Medicine, Division of Hospital Medicine and UHealth International, Department of Medicine, University of Miami Miller School of Medicine, USA

## Abstract

**Objectives:**

The primary outcome of this study is to assess the perspectives of Hospitalists on their workload and their perceived effects on patient care. The secondary outcomes are to evaluate the satisfaction of the Hospitalists with their compensation, quality of life, scholarship activity and promotion in their department and the support received to achieve this,

**Methodology:**

We developed a 49-question questionnaire. The questionnaire was based on (a) Oldenburg Burnout Inventory and (b) topics specific to census, compensation, academic support with desire for promotion, and the effects of workload on patient care and teaching. All questions were formatted with a 4-point Likert-type response scale. The questionnaires were distributed electronically using an online survey platform to all 32 of the Hospitalists at our institution.

**Conclusion:**

Each institution needs to do a self-assessment based on clinician feedback: Hospitalists workload, burn-out and satisfaction to reduce the high turnover rates and brevity of this role. From this study in this academic institution, the perspectives of Hospitalists revealed a high level of burn out (exhaustion and disengagement) and high assigned patient censuses that negatively impact their ability to deliver optimal patient care. Most Hospitalists reported lack of mentorship and inadequate time allocated for scholarly activity. The majority reported not having their input on decisions made by the administration that directly affect them. Most were unsatisfied with their compensation and the lack of PTO (paid time off). The majority would like to be promoted in this academic institution but feel unsupported to achieve this goal.

## Introduction

### Objectives

The primary outcome of this study is to assess the perspectives of Hospitalists on their workload and their perceived effects on patient care. The secondary outcomes are to evaluate the satisfaction of the Hospitalists with their compensation, quality of life, scholarship activity and promotion in their department and the support received to achieve this.

### Study Background

The Hospitalist model for inpatient care has significantly impacted inpatient medicine and is especially evident in academic medical centers.

Studies have shown that Hospitalists decrease the overall cost and length of stay for patients and readmission rates. Additionally, studies suggest superior teaching evaluations among Hospitalists, citing accessibility and provision of immediate feedback as strengths. [1]

The concept of the Hospitalist remains novel, with no standardized guidelines with regards to compensation or workload (number of encounters per day, complexity of patients and work hours, and the effect these factors may have on the quality of care of patients).

One study reported that the high workload of the Hospitalist contributes to incomplete discussions with patients and families, the ordering of unnecessary tests or procedures, delay in admissions or discharges, lower patient satisfaction, poorer handoffs, and other problems. For a recent survey posted on the-hospitalist.org, 51% of respondents picked “11 to 15” as the most appropriate patient census for a full-time Hospitalist.

From this study the researchers concluded that increasing the number of patients being seen or having high census numbers could paradoxically be increasing the costs of healthcare. [2] Another study concluded that increasing Hospitalist workload is associated with clinically meaningful increases in LOS and cost, suggesting the need for methods to mitigate the potential negative effects of increased Hospitalist workload on the efficiency and cost of care.

One study done to focus on productivity elevated the age-old question to the organizational finance department: “Is it better and financially more productive for the organization to lower the average starting census and to pay for the extra physician?” The answer was a resounding “Yes. [5]”

Compensation of Hospitalists vary widely across the nation. A report done with the 2020 State of Hospital Medicine (SoHM) partnering with the Medical Group Management Association (MGMA) provided data on Hospitalist compensation and productivity. The report offers significant and compelling evidence that Hospitalists continue to be compensated at rising rates due to the continued supply-and-demand mismatch and recognition of the overall value that Hospitalists generate rather than strictly the volume of their productivity.[3]

In an academic institution where Hospitalists teach medical students and residents, the number of patients that should be seen by Academic Hospitalist can become a contentious issue, focusing on the workload of the Hospitalist and the teaching and mentoring provided with high censuses. One study suggests that internal medicine clerkship student evaluations of Hospitalist faculty are negatively influenced by high clinical service intensity.^6^ The involvement in academia and the ability or desire for promotion, which depends on factors such as research and involvement in the University community, were all included in the study as most people are driven to become Hospitalists in academic centers by the desire to be involved in academics (teaching, education and research) rather than community hospitals. [6]

“Each institution needs to do a self-assessment based on clinician feedback. Is the workload manageable? What do their satisfaction surveys suggest? What are the turnover and burnout rates?” Ruth M. Kleinpell, PhD, RN, FAAN, FCCM, Professor of Nursing at Rush University Medical Center in Chicago and a nurse practitioner at Mercy Hospital and Medical Center. [7,8]

How many patients a Hospitalist should see in one day depends on many factors, including the Patient Case Mix Index (CMI), teaching or non-teaching service, admitting service versus consultative service, advanced practice providers, day-shift versus night-shift, observation versus regular admission patients, non-clinical duties, and hospital geography (where it can take a Hospitalist caring for 15 patients on 6 different nursing stations more time per day to manage than a hospitalist caring for 20 patients on a single nursing station and the patient demographic [4]).

Another factor to take into consideration of Hospitalist workload is the complexity of the patients. This study involves Hospitalists that are in a Health Network which includes an NCI designated cancer institute; thus, many admissions are complex cancer patients with complications of their malignancy or therapy.

Hospitalist burnout has become of growing concern with resultant high turnover rates; we experience this within our own institution. We thus thought it pertinent to incorporate an Oldenburg Burnout Inventory. “Burnout” is defined and measured as a work-related syndrome that is characterized by emotional exhaustion (i.e., a state of energy draining), cynicism (i.e., a sense of disengagement and gradual loss of concern about the contents or the recipients of one's work), and reduced professional efficacy (i.e., feelings of incompetence) that individuals experience in relation to their work. [9]

Nurse practitioners (NPs) are increasingly employed by hospital medicine groups and contribute to the care of the hospitalized adult patient. Prior research indicates NP hospitalists are empowered in their role. In this academic institution, the NPs admit and follow patients, but under the name of a Hospitalist and their admissions and discharges must be seen and signed by a Hospitalist, who is ultimately responsible for that patient. [10]

The goal of this study is to quantify the comfort level of the Hospitalist with their current census, to see if this impacts the quality of care for their patients, the desire and support in scholarly activities, their compensation, and their overall well-being at this institution.

This study will investigate the following interrelated questions as related to the Hospitalist:

1. The comfort level with the current census and the proposed census.
2. The effects that their current census has on overall patient care.
3. Their satisfaction with their involvement in scholarly activity, including teaching medical students.
4. Their satisfaction with their current compensation..
5. The favorability of having an ARNP.

From this study, we hope to achieve a better understanding of the above and implement and standardize changes that will lead to an overall and better outcome for the Hospitalists, patient care, teaching/mentoring at this academic institution.

## Methodology

We developed a 49-question questionnaire. The questionnaire was based on (a) Oldenburg Burnout Inventory [9] and (b) topics specific to census, compensation, academic support with desire for promotion, and the effects of workload on patient care and teaching. All questions were formatted with a 4-point Likert-type response scale. The questionnaires were distributed electronically using an online survey platform to all 32 of the Hospitalists (Faculty and Non-Faculty) in the University of Miami Health system, which consists of two academic inpatient facilities, a 560-bed tertiary hospital and a 40-bed cancer facility. Hospitalists working for less than one year were excluded from this study as we did not think they had sufficient time working in this institution to give valid answers. We collected responses from the Hospitalists in our inclusion criteria, consisting of 24, representing a 98% response rate. This study was exempted by our local IRB.

From this, potential conclusions were derived from the perspectives of the Hospitalists on that intended in the objective.

## Results

Most respondents have been Hospitalists for 1-3 years (eleven of the 24). Only two respondents had greater than ten years in the current role of a Hospitalist and seven respondents had between 5-10 years’ experience on the role. Twenty-one respondents were Faculty at our academic institution.

All Hospitalists agreed that the “seven days on and seven days off” work schedule was favorable. Fifteen Hospitalists preferred to start their work week on a Monday, whereas four Hospitalists opted for Tuesday or Wednesday.

Only two Hospitalists agreed that their financial compensation was adequate; most Hospitalists disagreed, with the majority indicating that a $50,000-$74,000 per annum pay increase would be reasonable and desired. See Table 1.

From the Oldenburg Burnout Inventory’s 16 questions, the median response rate was used to calculate the total score of 48 points, with a subtotal Disengagement of 23 points and Exhaustion subtotal of 23 points. Results indicate significant burnout amongst this group of Hospitalists. In addition, Hospitalists indicated missing 21-50% of important family events). See Table 2.

The following results are related to Hospitalists’ current census, views on census and ARNPs and suggestions for an appropriate patient census.

With regards to having an ARNP,43.48% of Hospitalists did not find this favorable and 30.43% of Hospitalist reported this being conditional, the most cited reason was: depends on ARNP and one Hospitalist mentioned having a structure and delegation of tasks.

Hospitalists reported currently having a census of 20-22 with an ARNP and a census of 16-18 without an ARNP. The two Non-Faculty personnel reported a census of 20.

With an ARNP, ten Hospitalists reported that an appropriate census would be 18; six Hospitalists thought that an appropriate cap would be 16; three Hospitalists answered 17 and three answered twenty.

One Hospitalist reported the current cap of 22 to be ideal. Table 3.

Without an ARNP, twenty Hospitalists indicated a cap of 12-14 as suitable. Nineteen of the hospitalists suggested that the cap should be adjusted for teams caring for > 50% progressive care or patients with malignancy or complications of chemotherapy. 100% of the Hospitalists suggested a lower cap with or without an ARNP. Table 3

96% of Hospitalists indicated that patient care is compromised by the high caps that they carry, taking the form of delayed discharges, communicating with patient and family (100%), paying closer attention to medical detail (100%), and the ability to utilize less consultative services (87%). Table 4

While twenty of the 22 faculty Hospitalists enjoyed teaching medical students, all 22 Hospitalists reported that their cap was not adjusted to accommodate teaching of these students and that this made their job more demanding. Table 5

Eighteen of the Hospitalists felt that they had no input in the decisions made by administration that directly impacts them, and 20 of the 22-faculty reported that there was inadequate support to perform scholarly activities (18 of whom cited that there was not enough time allocated to do so).

21 of the 22 faculty Hospitalists reported that they did not have a senior faculty mentoring them, with 18 of the 22 hoping to be promoted in the University in the future and 19 responded that there was “inadequate support to making promotion possible” Table 6.

When asked two things that they liked about being a Hospitalist, the responses included the patient mix; severity of illness and associated challenges; the process of inpatient work as patients come in sick and leave improved; patient interaction; peers; patient mix; taking care of sick patients; schedule; and comprehensive exposure to patients in their complexity.

When asked what changes they would like to see implemented, the Hospitalists“ responses were: mandatory vacation; lower cap or higher (at least current fair market value) compensation; salary increase to fair market value; lower caps and improved the communication with specialists Table 7.

## Discussion

The Hospitalist model for inpatient care has significantly impacted inpatient medicine and is clearly more evident in academic medical centers.

The survey conducted among Hospitalists in our academic medical center revealed high levels of burnout (disengagement and exhaustion) using the Oldenburg Burnout Inventory’s 16 questions, with a total score of 48 points, with a subtotal Disengagement of 23 points and Exhaustion subtotal of 23 points. In addition, Hospitalists indicated missing 21-50% of important family events

The overwhelming majority of Hospitalists in this institution indicated that their high census compromised patient care with longer length of stays, inability to pay closer attention to medical detail, and the inability to communicate with the patients and their families as the Hospitalist would wish to. The overwhelming majority reported a desire for a lower cap with or without an ARNP.

43.48% of Hospitalists did not find working with an ARNP to be favorable and 30.43% of Hospitalist reported working with an ARNP is conditional, the most cited reason was: “depends on the specific ARNP”.

All Hospitalists agreed that it was more demanding to have medical students and, due to the lack of accommodations for this, such as a lowered census. This made having medical students on their service, an activity that they enjoy, an added burden to their workload.

Most Hospitalists also reported not enough time for scholarly activity. Faculty Hospitalists also indicated lack of mentorship, and though the vast majority wanted to be promoted they did not feel supported in their desire to do so.

Most Hospitalists reported that the administration made decisions without their input and that they should have 2-3 weeks PTO (paid time off) and that they were receiving a lower than preferred compensation.

This study was to gain the perspectives of the Hospitalists. The results are not surprising considering the literature review in the study’s background.

This high level of dissatisfaction leads to a high turnover rate and brevity in the position.

## Conclusion

Each institution needs to do a self-assessment based on clinician feedback on Hospitalists workload<burn-out and satisfaction to reduce the high turnover rates and brevity of this role. From this study in this academic institution, the perspectives of Hospitalists revealed a high level of burn out (exhaustion and disengagement) and high assigned patient censuses that negatively impact their ability to deliver optimal patient care. Most Hospitalists reported lack of mentorship and inadequate time allocated for scholarly activity. The majority reported not having their input on decisions made by the administration that directly affects them. Most were unsatisfied with their compensation and the lack of PTO (paid time off). The majority would like to be promoted in this academic institution but feel unsupported to achieve this goal.

## Figures and Tables

**Table 1:** Hospitalists Schedule and Compensation.


**Q1. How long have you been a Hospitalist?**		
Answer Choices	Responses	
<1 year	0.00%	0
1-3 years	47.83%	11
3-5 years	13.04%	3
5-10 years	30.43%	7
>10 years	8.70%	2
	**Answered**	**23**
	**Skipped**	**1**
		
**Q2. Are you Faculty/ Voluntary Faculty or House staff Physician**?		
Answer Choices	Responses	
Faculty	87.50%	21
Voluntary Faculty	0.00%	0
Staff Physician (non-Faculty)	12.50%	3
	**Answered**	**24**
	**Skipped**	**0**
		
**Q3. Do you find the seven days on and seven days off schedule favorable?**		
Answer Choices	Responses	
Strongly agree	50.00%	12
Agree	50.00%	12
Disagree	0.00%	0
Strongly disagree	0.00%	0
	**Answered**	**24**
	**Skipped**	**0**
		
Q4. **What day of the week would you prefer to start your service week on?**		
Answer Choices	Responses	
Monday	62.50%	15
Tuesday	16.67%	4
Wednesday	16.67%	4
Thursday	0.00%	0
Friday	4.17%	1
Saturday	0.00%	0
Sunday	0.00%	0
	**Answered**	**24**
	**Skipped**	**0**
Q5. **Do you think you should have an additional 2–3-week PTO (paid time off) included in your schedule?**		
Answer Choices	Responses	
Strongly agree	86.96%	20
Agree	8.70%	2
Disagree	4.35%	1
Strongly disagree	0.00%	0
	**Answered**	**23**
	**Skipped**	**1**
Q6. **Is the financial compensation being satisfactory?**		
Answer Choices	Responses	
strongly agree	0.00%	0
Agree	8.70%	2
Disagree	43.48%	10
Strongly disagree	47.83%	11
	**Answered**	**23**
	**Skipped**	**1**
Q7. **How much more would you consider to be an appropriate annual compensation?**		
Answer Choices	Responses	
>$100,000.00	13.04%	3
$75,000- $99,000.00	13.04%	3
$50,000-$74,000.00	47.83%	11
$25,000-$49,000.00	17.39%	4
<$25,000.00	0.00%	0
Compensation adequate	8.70%	2
Other (please specify)	0.00%	0
	**Answered**	**23**
	**Skipped**	**1**

**Table 2:** Oldenburg Burnout Inventory.

Q9. **There are days when I feel tired before I arrive at work**		
Answer Choices	Responses	
Strongly agree	56.52%	13
Agree	39.13%	9
Disagree	0.00%	0
Strongly disagree	4.35%	1
	Answered	23
	Skipped	1
		
Q10. **It happens more and more often that I talk about my work in a negative way**		
Answer Choices	Responses	
Strongly agree	43.48%	10
Agree	39.13%	9
Disagree	17.39%	4
Strongly disagree	0.00%	0
	Answered	23
	Skipped	1
		
Q11. After work, I tend to need more time than in the past to relax and feel better		
Answer Choices	Responses	
Strongly agree	60.87%	14
Agree	17.39%	4
Disagree	17.39%	4
Strongly disagree	4.35%	1
	Answered	23
	Skipped	1
		
Q12. **I can tolerate the pressure of my work very well**		
Answer Choices	Responses	
Strongly agree	4.35%	1
Agree	47.83%	11
Disagree	39.13%	9
Strongly disagree	8.70%	2
	Answered	23
	Skipped	1
		
**Q13. Lately, I tend to think less at work and do my job almost mechanically**		
Answer Choices	Responses	
Strongly agree	26.09%	6
Agree	56.52%	13
Disagree	17.39%	4
Strongly disagree	0.00%	0
	Answered	23
	Skipped	1
		
Q14**. I find my work to be a positive challenge**		
Answer Choices	Responses	
Strongly agree	4.35%	1
Agree	47.83%	11
Disagree	39.13%	9
Strongly disagree	8.70%	2
	Answered	23
	Skipped	1
		
Q15. **During my work, I often feel emotionally drained**		
Answer Choices	Responses	
Strongly agree	47.83%	11
Agree	34.78%	8
Disagree	13.04%	3
Strongly disagree	4.35%	1
	Answered	23
	Skipped	1
		
Q16. **Over time, one can become dis-connected from this type of work**		
Answer Choices	Responses	
Strongly agree	30.43%	7
Agree	34.78%	8
Disagree	21.74%	5
Strongly disagree	13.04%	3
	Answered	23
	Skipped	1
Q17. **After working, I have enough energy for my leisure activities**		
Answer Choices	Responses	
Strongly agree	0.00%	0
Agree	17.39%	4
Disagree	52.17%	12
Strongly disagree	30.43%	7
	Answered	23
	Skipped	1
		
Q18. **Sometimes I feel sickened by my work tasks**		
Answer Choices	Responses	
Strongly agree	26.09%	6
Agree	39.13%	9
Disagree	34.78%	8
Strongly disagree	0.00%	0
	Answered	23
	Skipped	1
		
Q19. **After my work, I usually feel worn out and weary**		
Answer Choices	Responses	
Strongly agree	47.83%	11
Agree	43.48%	10
Disagree	8.70%	2
Strongly disagree	0.00%	0
	Answered	23
	Skipped	1
		
Q20. **This is the only type of work that I can imagine myself doing**		
Answer Choices	Responses	
Strongly agree	4.35%	1
Agree	21.74%	5
Disagree	60.87%	14
Strongly disagree	13.04%	3
	Answered	23
	Skipped	1
		
Q21. **Usually, I can manage the amount of my work well**		
Answer Choices	Responses	
Strongly agree	4.35%	1
Agree	65.22%	15
Disagree	30.43%	7
Strongly disagree	0.00%	0
	Answered	23
	Skipped	1
		
Q22. **I feel more and more engaged in my work**		
Answer Choices	Responses	
Strongly agree	0.00%	0
Agree	17.39%	4
Disagree	69.57%	16
Strongly disagree	13.04%	3
	Answered	23
	Skipped	1
		
Q23. **When I work, I usually feel energized**		
Answer Choices	Responses	
Strongly agree	0.00%	0
Agree	21.74%	5
Disagree	65.22%	15
Strongly disagree	13.04%	3
	Answered	23
	Skipped	1
		
Q24**. Are you missing important family events**		
Answer Choices	Responses	
< 20% of the time	21.74%	5
21-50% of the time	47.83%	11
>51% of the time	30.43%	7
	Answered	23
	Skipped	1

**Table 3:** Census and ARNP.

Q25. Is working with an ARNP favorable to you?		
Answer Choices	Responses	
Strongly agree	8.70%	2
Agree	17.39%	4
Disagree	26.09%	6
Strongly Disagree	17.39%	4
Other (please specify)	30.43%	7
	**Answered**	**23**
	**Skipped**	**1**
		
**Other (please specify)**		
Depends on the specific ARNP		
Yes, but the structure MUST be different in terms of delegating tasks. There is no proper structure		
No		
It depends on the ARNP you are working with		
If the ARNP is experienced and knowledgeable then it can work but not in the current system		
Depends on ARNP		
Depends on the ARNP		
Q26. **With an ARNP, how many patients is the current cap?**		
**Answered**		**21**
**Skipped**		**3**
		
Census		Number of Hospitalists
22		19
n/a		1
20		1
		
Q27. **Without an ARNP, how many patients is the current cap?**		
**Answered**		**21**
**Skipped**		**3**
		
Census		Number of Hospitalists
16		19

18		1
n/a		1
**Q28. What do you think the cap should be with an ARNP?**		
**Answered**		**22**
**Skipped**		**2**
		
Census		Number of Hospitalists
16		20
16-18		1
n/a		1
Q29. **What do you think the cap should be without an ARNP?**		
**Answered**		22
**Skipped**		2
		
Census		Number of Hospitalists
10		9
12		1
13		7
14		3
16		1
**Q30. Do you think teams caring for more than 50% of patients admitted to Progressive Care or for malignancy or complications of malignancy or chemotherapy, should have a lower cap adjustment than other teams?**		Responses
**Answered**		**23**
**Skipped**		**1**
		
Yes		19
No		4
Q31**. If ‘Yes’ to #21, what cap would you suggest with an ARNP?**		
**Answered**		**17**
**Skipped**		7
Census		Number of Hospitalists
10		1
12		6
14		4
15		1
16		2
18		2
20		1
Q32. **If ‘Yes’ to #21, what cap would you suggest without an ARNP?**		
**Answered 16**		
**Skipped 8**		
Census		Number of Hospitalists
8		1
10		4
12		4
13		1
14		4

**Table 4:** Patient Care.

Q33. **Do you think ‘Patient Care: Delayed Discharges’ could be prevented with lower census/cap?**		
Answer Choices	Responses	
Yes	95.65%	22
No	4.35%	1
	**Answered**	**23**
	**Skipped**	**1**
		
Q34. Do you think ‘Patient Care: Communicating with Patient and Family’ would be enhanced with a lower census?		
Answer Choices	Responses	
Yes	100.00%	23
No	0.00%	0
	**Answered**	**23**
	**Skipped**	**1**
		
Q35. Do you think that ‘Patient Care: Closer Attention to Medical Detail’ would be enhanced with a lower census?		
Answer Choices	Responses	
Yes	100.00%	23
No	0.00%	0
	**Answered**	**23**
	**Skipped**	**1**
Q36. Do you think that Patient Care: 'utilizing less consultative services'. would be enhanced by a lower census?		
Answer Choices	Responses	
Yes	86.96%	20
No	13.04%	3
	**Answered**	**23**
	**Skipped**	**1**
Q37. In the last twelve months, have you had a medical student?		
Answer Choices	Responses	
less than 25% of the time	43.48%	10
25-50% of the time	26.09%	6
50-75% of the time	13.04%	3
>75% of the time	8.70%	2
Never	8.70%	2
	**Answered**	**23**
	**Skipped**	**1**

**Table 5:** Teaching.

**Q38. Do you enjoy teaching medical students?**		
Answer Choices	Responses	
Strongly agree	52.17%	12
Agree	34.78%	8
Disagree	4.35%	1
Strongly disagree	8.70%	2
	**Answered**	**23**
	**Skipped**	**1**
		
Q39. Is your cap adjusted to accommodate teaching of the medical student?		
Answer Choices	Responses	
Yes	4.35%	1
No	95.65%	22
	**Answered**	**23**
	**Skipped**	**1**
		
**Q40. Does having a medical student make your job more demanding?**		
Answer Choices	Responses	
Yes	95.65%	22
No	4.35%	1
	**Answered**	**23**
	**Skipped**	**1**
		
**Q41. Is working with residents is favorable to you?**		
Answer Choices	Responses	
Strongly agree	39.13%	9
Agree	43.48%	10
Disagree	13.04%	3
Strongly disagree	4.35%	1
	**Answered**	**23**
	**Skipped**	**1**

**Table 6:** Scholarly activity, mentoring, promotion, and administration.

**Q42. Do you feel that you have an input in the decisions made by administration that directly impacts your work?**		
Answer Choices	Responses	
Yes	21.74%	5
No	78.26%	18
	**Answered**	**23**
	**Skipped**	**1**
**Q43. Do you think there is enough support for you to perform scholarly activities?**		
Answer Choices	Responses	
Yes	13.04%	3
No	86.96%	20
	**Answered**	**23**
	**Skipped**	**1**
**Q44. Do you think there is enough time allocated for scholarly activities?**		
Answer Choices	Responses	
Yes	21.74%	5
No	78.26%	18
	**Answered**	**23**
	**Skipped**	**1**
**Q45. Do you have a senior faculty mentoring you?**		
Answer Choices	Responses	
Yes	8.70%	2
No	91.30%	21
	**Answered**	**23**
	**Skipped**	**1**
**Q46. Do you wish to be promoted in this University in the future?**	Responses	
Yes	78.26%	18
No	4.35%	1
N/A	17.39%	4
	**Answered**	**23**
	**Skipped**	**1**
**Q47. Do you believe you are being given the support for promotion to be possible?**		
Answer Choices	Responses	
Yes	17.39%	4
No	82.61%	19
	**Answered**	**23**
	**Skipped**	**1**

**Table 7:** Hospitalists: what they currently enjoy and changes they would implement.

**Q48. What are two things you enjoy about being a Hospitalist?**	
Teaching	
Patient care	
Caring for patients with a wide variety of clinical	
Variety of pathology	
Providing direct care for acutely ill patients	
variety of patients - ability to see the wide gamut of internal medicine	
Not being restricted to just one organ related pathology	
7 on 7 off	
Type of patient encounter	
Schedule	
To be able to handle different pathologies	
Schedule	
I’m Nocturnist I like straight forward job	
My peers in the division	
Taking care of sick patients	
The patient mix	
Schedule having 7 days off in a row.	
Severity of illness and associated challenges	
I enjoy the process of inpatient work as patients come in sick and leave improved	
Patient interaction	
Comprehensive exposure to patients in their complexity	
Q49. **If there were two changes you could implement in your current job as a Hospitalist, what would they be?**	
**Answered**	**20**
**Skipped**	**4**
A clear pathway to develop academic hospitalists	
Lower the patient census	
Lower the cap	
2 weeks of PTO/vacation (not on our off weeks)	
increased structural support including but not limited to the development of the department as whole, access to research support, administrative support, financial restructuring, and development with models considering how other academic systems work, workflow support (having dedicated discharge planning teams supporting academic teams, along with pharmacist, SW, case management, etc.).	
Census control, compensation	
PTO/higher pay	
There needs to be frequent mtgs with CM and RNs educating them to first off communicate with one another, so we do not get 4-5 phone calls from bedside RN, charge RN, dc planner and CM about the same thing. Pls know when it is appropriate to call a physician. I have received calls asking about social issues that are completely non-medical requiring me to stop handling a serious medical problem to deal w/ social issues.	
Less handoff/sign-out (multiple providers within 1 week)	
Less patient	
Support from admin including more frequent communication and asking for input before instituting changes	
Decrease cap	
mandatory vacation	
Lower cap or higher (at least current fair market value) compensation	
Salary increases to fair market	
Lower caps	
Eliminate floor triage	
Improve the communication with specialists	
Start work week on Tuesday	
Shared decision making with transparency	
Census control and focus on real quality outcomes	
Get more support for scholarly/educational activities	
Start working on Tuesday instead of Monday	
Decrease census cap or increase salary (if caps are not decreased)	
education to administration and the department/HR regarding salaries for Hospital Medicine based on current and ongoing responsibilities placed on the division, complexity of care of our growing census of patients, and the ever-increasing workload placed on hospital medicine department, amid inflation and increasing prices in Miami.	
Having the ability to progress in career or being given equal opportunity to all the staff w both teaching and non-teaching irrespective of how many years of your experience is with UM	
Options for more than 1 week vacation block	
Geographic location of patients/teams	
Actual vacation days	
Get more support staff	
Better nursing support	
Stability of chair and administrators.	
collective bargaining agreement with administration about decisions	

